# Green Port Development — Welche Rolle kommt Häfen bei der Erreichung der Klimaziele zu?

**DOI:** 10.1007/s10273-021-2897-2

**Published:** 2021-04-17

**Authors:** Dörte Nitt-Drießelmann, Jan Wedemeier

**Affiliations:** 1 HWWI, Fahrenheitstr. 1, 28359 Bremen, Deutschland; 2 HWWI, Oberhafenstraße 1, 20097 Hamburg, Deutschland

**Keywords:** R10, R11, Q2

## Abstract

Ein Großteil des europäischen Warentransports erfolgt mit dem Schiff. Häfen, die die Infrastruktur für maritime Aktivitäten bereitstellen, sind wichtige Akteure im maritimen Sektor und von hoher Bedeutung für die europäische Wirtschaft. Um internationale Klimaabkommen erfüllen zu können, müssen Hafenanlagen künftig nachhaltiger und emissionsärmer betrieben werden, damit an den jeweiligen Standorten die lokalen CO_2_-Fußabdrücke reduziert werden können.

## Abstract (en)

Maritime activities are an important part of the European economy as many goods are transported by ship. Ports are important players in the maritime sector because they provide the infrastructure for many maritime activities. Therefore, the potential impact of the port on the local carbon footprint should not be underestimated.

Im Jahr 2019 wurden 46,0 % der EU-28-Exporte und 56,2 % der EU-28-Importe verschifft (Eurostat, 2020).[1] Die Europäische Kommission erwartet aufgrund der sich verstärkenden globalen wirtschaftlichen Integration einen weiteren Anstieg des Seeverkehrs und des Seehandels (Europäische Kommission, 2019a). Diese Einschätzung bleibt trotz des COVID-19-Schocks, der zwischen März und Juni 2020 zu einem Rückgang des beförderten Warenvolumens auf See führte, und trotz der Entstehung möglicher neuer Handelsbarrieren unverändert bestehen (Schumacher et al., 2020). Es ist zu erwarten, dass die Emissionen im internationalen Seeverkehr ohne zusätzliche politische Maßnahmen im Vergleich zu 2015 bis zum Jahr 2035 um 23 % zunehmen könnten (ITF, 2018). Auf globaler Ebene gehen Schätzungen zu den Seeverkehrsemissionen von Anteilen zwischen 2,5 % und 3,6 % an den gesamten Treibhausgasen aus (IMO, 2014; IMO, 2019; Eurostat 2021). Der Anteil des Seeverkehrs in Bezug auf die weltweiten Stickoxid- und Schwefeldioxidemissionen liegt bei rund 13 % bis 15 % (Winkel et al., 2016).

Zwischen 2008 und 2018 sind die Luftemissionskonten in der EU 28 insgesamt leicht zurückgegangen, wobei nach einer durch die Finanz- und Währungskrise 2008/2009 bedingten stärkeren Abnahme ein leichter Anstieg in den darauffolgenden Jahren zu verzeichnen gewesen ist. Aufgrund der COVID-19-Pandemie wird auch für die Jahre 2020 und 2021 ein Einbruch in den Luftemissionskonten erwartet. Der Anteil der Verkehrsleistungen (Landverkehrs-, Schifffahrts- und Luftfahrtleistungen) an allen Luftemissionen aus wirtschaftlichen Aktivitäten (NACE-Codes) einschließlich aller Haushalte sanken zwischen 2008 und 2010 von 7,0 % auf 5,5 % und haben sich seitdem bis 2018 kontinuierlich auf 7,4 % erhöht. Unter den Verkehrsleistungen entfiel 2018 mit 46,5 % der größte Anteil an Luftemissionen auf die Luftfahrtleistungen — das entspricht einem Plus von 12,8 Prozentpunkten gegenüber 2008. Der Anteil der Luftemissionen in der Schifffahrt an den gesamten Luftemissionen der Verkehrsleistungen sank hingegen um 8 Prozentpunkte – von 39,5 % (2008) auf 31,5 % (2018) (Eurostat, 2021) (vgl. Abbildung 1).[2] In der absoluten Betrachtung gingen die Emissionen in der Schifffahrt zwischen 2008 und 2018 um 43,4 Mio. Tonnen zurück.

1 Der Artikel basiert auf Forschungsergebnissen des EU-Projekts „DUAL Ports – Developing Low carbon Utilities, Abilities and potential of regional entrepreneurial Ports“ (INTERREG North Sea Region Programme). Das EU-finanzierte Projekt DUAL Ports zielt darauf ab, regionale Häfen durch innovative Hafeninvestitionen zu dekarbonisieren, die zur Minimierung von Emissionen beitragen (www.dualports.eu). Die geringere Größe der regionalen Häfen ermöglicht es, potenzielle innovative Maßnahmen und Technologien zu testen, um diese dann – bei einer Marktdurchsetzung – EU-weit zu implementieren.

2 In den EU-28-Luftemissionskonten (Restgas- und Partikelmaterialien) werden Emissionen erfasst, die sich aus den (wirtschaftlichen) Aktivitäten aller ansässigen Einheiten (Emissionen von in der übrigen Welt, von gebietsansässigen Einheiten (EU 28) sowie im Wirtschaftsgebiet (EU 28) von gebietsfremden Einheiten) ergeben, unabhängig davon, wo diese Emissionen tatsächlich geografisch auftreten. Luftemissionskonten basieren ebenfalls auf dem Aufenthaltsprinzip. Natürliche (Aus-)Strömungen von gasförmigen und teilchenförmigen Restmaterialien werden in den Berechnungen ausgeschlossen (z.B. Waldbrände, aber auch aus z.B. Landnutzungsänderungen) (Eurostat, 2021).

**Dörte Nitt-Drießelmann** ist Senior Researcher am Hamburgischen WeltWirtschaftsInstitut (HWWI).

**Dr. Jan Wedemeier** ist Senior Researcher am HWWI in Bremen und Leiter des Forschungsbereichs „Ökonomie der Städte und Regionen“.

Abbildung 1
**Luftemissionsrechnungen insgesamt zu Emissionsinventaren insgesamt, EU-28**

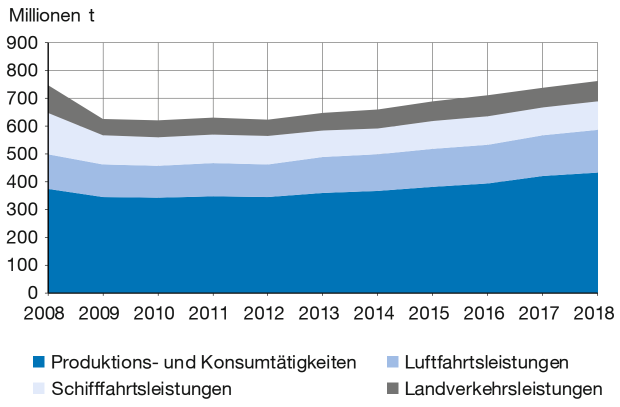

Treibhausgase (THG) ($CO_2$, $N_2O$ in $CO_2$-Äquivalent, $CH_4$ in $CO_2$-Äquivalent, HFC in $CO_2$-Äquivalent, PFC in $CO_2$-Äquivalent, $SF_6$ in $CO_2$-Äquivalent, $NF_3$ in $CO_2$-Äquivalent), Emissionen in der übrigen Welt, von gebietsansässigen Einheiten (EU-28) sowie im Wirtschaftsgebiet (EU-28) von gebietsfremden Einheiten.

Quellen: Eurostat (2021), HWWI.

## Europäische Initiativen zur Reduzierung der Schifffahrtsemissionen

Schiffe werden überwiegend mit fossilen Brennstoffen betrieben. Diese emittieren neben $CO_2$ und Schwefeldioxid ($SO_2$) auch weitere Stoffe wie Stickstoffoxide ($NO_x$) oder Particulate Matter (PM), die sich negativ auf die Umwelt und die menschliche Gesundheit auswirken. Schweröl (HFO/IFO) und Marinedieselöl (MDO) machen 90 % des Energieverbrauchs der internationalen Schifffahrt aus (Fenhann, 2017). Sie werden überwiegend aus Abfallprodukten der Raffinierung und Dieselöl gemischt.

Mit dem „White Paper 2011" hat die Europäische Kommission einen Fahrplan mit Initiativen zur Verbesserung der Mobilität sowie zur Reduzierung der $CO_2$-Emissionen im Verkehr um 60 % bis 2050 aufgezeigt (Europäische Kommission, 2011; Froese et al., 2019). Die wichtigsten Ziele im Bereich der Schifffahrt sind die Reduzierung der Schifffahrtsemissionen um mindestens 40 % sowie eine 50-prozentige Verlagerung des Personen- und Güterverkehrs über mittlere Entfernung von der Straße auf die Schiene und den Wasserweg. Zur Reduzierung der Treibhausgasemissionen hat die EU darüber hinaus eine Strategie zur schrittweisen Einbeziehung der Meeresemissionen in die EU-Politik verabschiedet (Europäische Kommission, 2015). Die Verordnung 2015/7575 zur Überwachung, Berichterstattung und Überprüfung der Kohlendioxidemissionen aus dem Seeverkehr (EU-MRV-Verordnung) regelt, dass Reedereien relevante Informationen zu Emissionen erfassen, die sich aus den Schiffsfahrten zu und von Häfen sowie innerhalb der Häfen des Europäischen Wirtschaftsraums (EWR) ergeben (Europäische Kommission, 2015). Diese Verpflichtungen für Reedereien begannen 2017 mit der Erstellung und Übermittlung von Überwachungsplänen an akkreditierte Prüfer:innen. Die ersten Kontrollen des Kraftstoffverbrauchs, der $CO_2$-Emissionen und der Energieeffizienz folgten 2018. Die ersten Emissionsberichte waren 2019 fällig (Froese et al., 2019).

## Häfen: Multifunktionale Akteure ohne festgelegte EU-Emissionsziele

Häfen bilden einen entscheidenden Knotenpunkt in der globalen Transportkette (Bergqvist und Monios, 2019). Sie stellen an ihren Standorten die Infrastruktur für die meisten maritimen Aktivitäten, aber auch für nicht maritime Aktivitäten, wie die Stromerzeugung oder die industrielle Produktion, bereit. Diese Multifunktionalität hat zur Folge, dass die Dekarbonisierung von Häfen ein breites Tätigkeitsfeld umfasst. Nachhaltige Häfen – Green Ports – müssen laut World Ports Climate Initiative (2010) sowohl in mobilen als auch in stationären Quellen (d. h. Supra-, Infrastruktur, Maschinen und Fahrzeuge) ressourceneffizient sein. Sie sollen Emissionen reduzieren und gleichzeitig effizient arbeiten. Die Internationale Seeschifffahrts-Organisation (IMO) folgte dem EU-System, indem sie ein IMO-Datenerfassungssystem einrichtete, das im März 2018 in Kraft trat und 2019 mit der Erfassung der Kraftstoffverbrauchsdaten begann. Seit 2019 müssen Schiffe, die in EWR-Häfen anlaufen, sowohl gemäß der EU-MRV-Verordnung als auch entsprechend des IMO-Datenerfassungssystems Bericht erstatten (Europäische Kommission, 2019b).

Häfen sind Umschlagplätze, die nach nationalen Vorschriften betrieben werden und den EU-Statuten für industrielle Infrastruktur und Betriebe entsprechen müssen. Derzeit gibt es keine Verordnungen der EU-Kommission, die sich explizit mit dem $CO_2$-Fußabdruck von Häfen befassen oder Einsparziele festlegen, die erforderlich wären, um zur Erreichung des Treibhausgasemissionsziels bis 2050 beizutragen (Froese et al., 2019). Formal und rechtlich gesehen ist ein Hafen für $CO_2$-Emissionen innerhalb, nicht jedoch für diejenigen außerhalb des Hafengebiets verantwortlich, obwohl sich die potenziellen Auswirkungen eines Hafens auf den lokalen $CO_2$-Fußabdruck nicht auf die Hafenprozesse beschränken. Häfen können dazu beitragen, die Emissionen auch außerhalb ihres Hafengebiets einzudämmen, indem sie beispielsweise entsprechende Anreize für die Anbieter der über Straße oder Schiene laufenden Hinterlandtransporte setzen sowie (grünen) Landstrom bereitstellen oder Flüssigerdgas-Bunker organisieren, damit Seeschiffe ihren $CO_2$-Fußabdruck verringern können.

### Maßnahmen und Priorisierung der Umweltrisiken durch Seehäfen

Die European Sea Port Organisation (ESPO) vertritt Hafenbehörden, Hafenverbände und Verwaltungen der Seehäfen der Mitgliedstaaten der Europäischen Union und Norwegens. Für den Umweltbericht der ESPO befragt die Organisation Vertreter:innen von EU-Häfen, welche Umweltrisiken sie identifiziert und überwacht haben, um eine Liste der Prioritäten für Maßnahmen und Einhaltung der Risiken erstellen zu können (ESPO, 2020).[3]

Neben Antworten zu verschiedenen ökologischen Managementindikatoren, wie z. B. „Hat der Hafen eine Umweltpolitik?“, enthält der Report Auskünfte zu den Umweltüberwachungen der Seehäfen in Bezug auf Abfall, Energieverbrauch, Wasser-, Luft-, Sediment- und Bodenqualität, $CO_2$-Fußabdruck, Meeresökosystemen und terrestrischen Lebensräumen. In unterschiedlichen zeitlichen Abständen priorisierten die Vertreter:innen der Seehäfen darüber hinaus Kriterien für ökologische Maßnahmen. Basierend auf den Antworten der befragten Leitungsgremien ergibt sich eine Rangfolge der wichtigsten Umweltprioritäten (vgl. Tabelle 1). Die Luftqualität hat seit 2013 für die Häfen oberste Priorität. Der Energieverbrauch, 2017 noch auf Rang 10, belegt 2020 den zweiten Rang, gefolgt vom Lärm, der seit 2013 jeweils unter den Top 3 zu finden ist. Der Klimawandel erscheint 2013 erstmals unter den Top 10 (ESPO, 2020).

Tabelle 1
**Top 10 Umweltprioritäten der Häfen**

| | 2004 | 2009 | 2013 | 2016 | 2017 | 2018 | 2019 | 2020 |
|---|---|---|---|---|---|---|---|---|
| Luftqualität | 6 | 2 | 1 | 1 | 1 | 1 | 1 | 1 |
| Energieverbrauch | : | : | : | : | 10 | 7 | 3 | 2 |
| Lärm | : | 7 | 3 | 1 | 2 | 2 | 2 | 3 |
| Nachbarschaft | 5 | 1 | 4 | 3 | 3 | 3 | 4 | |
| Schiffsabfälle | : | 6 | 6 | 4 | 8 | 4 | 5 | 5 |
| Hafenentwicklung (landseitig) | : | : | 5 | 6 | 9 | 5 | 6 | 6 |
| Klimawandel | : | : | 10 | 8 | 4 | 8 | 10 | 7 |
| Wasserqualität | 1 | 3 | 2 | 5 | 6 | 10 | 7 | 8 |
| (Nass-)Baggertätigkeiten | 2 | 4 | 7 | 10 | 5 | 9 | 9 | 9 |
| Müll/Hafenabfälle | 9 | 10 | 9 | 7 | 7 | 6 | 8 | 10 |
| (Nass-)Ausbaubaggergut | 8 | : | : | : | : | : | : | : |
| Staub | 3 | 5 | : | : | : | : | : | : |
| Gefährliche Fracht | 4 | 8 | 8 | 9 | : | : | : | : |
| Bunkern | 7 | : | : | : | : | : | : | : |
| Hafenentwicklung (wasserseitig) | : | 9 | : | : | : | : | : | : |
| Schiffsentladung (Bilge) | 10 | : | : | : | : | : | : | : |

Anmerkung: Prioritäten der Häfen nach Jahr.

Quellen: ESPO (2020); HWWI.

### Regionale Häfen: Anlegemanöver für Spezialisten

Neben den großen Seehäfen wie Bremerhaven, Hamburg und Rotterdam finden sich in Europa viele kleinere Häfen mit regionalwirtschaftlicher Bedeutung. Diese EU-Häfen stehen vor zwei spezifischen Herausforderungen. Einerseits sind die finanziellen Ressourcen der regionalen Häfen häufig begrenzt, andererseits müssen sie im Wettbewerb der Regionalhäfen bestehen können. Dies geschieht in der Regel durch Spezialisierung auf Spezialverkehre und Projektladungen, deren Wachstumschancen durch die zunehmende internationale Arbeitsteilung und entsprechende Neupositionierungen von Wertschöpfungsketten ganzer Industrien begünstigt werden. Regionalhäfen sind in solchen Netzwerken als Knotenpunkte für die Zusammenführung von Vorleistungen, aber auch für eine mögliche Endfertigung besonders geeignet (Lutzky, 2012).

Beide Herausforderungen implizieren, dass regionale Häfen nach innovativen Maßnahmen suchen müssen, um sich im Wettbewerb positionieren zu können (Froese et al., 2019). Wie bereits der ESPO-Bericht zeigt, sind Umweltthemen im Kontext der regionalen Hafenentwicklung von hoher Bedeutung (vgl. Tabelle 1). Kleinere Häfen, von denen viele bereits an der Hauptinitiative für grüne Häfen beteiligt sind, sollten somit die Umsetzung von Umweltschutzmaßnahmen in Betracht ziehen. Auch hier besteht ein enormes Potenzial für eine positive Entwicklung und Beteiligung (DUAL Ports, 2020).

### Schlussfolgerung

Zusammenfassend zeigt sich, dass der Energieverbrauch in Form fossiler Brennstoffe die Hauptquelle für Emissionen im Seeverkehr darstellt. Daher bietet der Ersatz von fossilen durch alternative, weniger emissionsintensive Energieträger das größte Potenzial zur Dekarbonisierung des Seeverkehrs. Nicht nur große Seehäfen, auch kleinere regionale, spezialisierte Häfen können hierzu ihren Beitrag leisten. Da Häfen an ihren Standorten nicht nur die Infrastruktur für maritime Aktivitäten, sondern auch für wirtschaftliche Tätigkeiten wie Stromerzeugung oder industrielle Produktion bereitstellen, umfasst die Dekarbonisierung von Häfen ein breites Tätigkeitsfeld. Green Ports – nachhaltige Häfen – sollten sowohl bei mobilen als auch bei stationären Emittenten ressourceneffizient ar-

3 2020 nahmen 97 Häfen an der Umfrage teil, wobei die größte Teilnehmerzahl der Umfrage aus Großbritannien kommt (15,5 %), gefolgt von Spanien (14,4 %) sowie Frankreich und Deutschland (je 10,3 %).

beiten und dafür ein intelligentes Management betreiben. Da sich die potenziellen Auswirkungen eines Hafens auf den lokalen $CO_2$-Fußabdruck nicht ausschließlich auf Hafenprozesse beschränken, sollten Häfen durch geeignete Anreizsysteme auch zu der Emissionsreduzierung außerhalb ihres Hafengebiets beitragen. So können sie beispielsweise Anbieter von Hinterlandtransporten dazu verpflichten, gewisse Umweltauflagen einzuhalten, oder die Emissionen durch Seeschiffe während der Ladung und Löschung der Waren durch die Bereitstellung von grünem Landstrom innerhalb des Hafengebiets reduzieren.

Aus internationaler Sicht kann die Dekarbonisierung durch $CO_2$-Emissionszertifikate oder $CO_2$-Steuern erreicht werden. Zwar hat die EU mit dem EU-Emissionshandelssystem (EU-EHS) bereits ein $CO_2$-Handelssystem eingerichtet, das jedoch vor allem energieintensive Industrien und die Luftfahrt betrifft, sodass damit etwa 38 % der europäischen Treibhausgasemissionen erfasst werden (Europäische Kommission, 2020). Die europaweiten Emissionen, die unter dem EU-EHS gedeckelt werden, konnten seit Einführung des EU-EHS 2005 um rund 35 % gesenkt werden (Umweltbundesamt, 2020). Offen ist, ob dies auf das Emissionssystem unmittelbar zurückzuführen ist oder z. B. auf strukturellem Wandel basiert. Um Emissionen weiter zu senken, sollte ein EU-weites System für alle (NACE-)Sektoren in Betracht gezogen und die Zahl der gehandelten Zertifikate verringert werden. Ein anderer erfolgversprechender Weg könnte in der Einführung einer Pigou-Steuer als ein steuerliches Instrument liegen. Hierbei würden durch ökonomische Aktivitäten entstehende, negative externe Effekte für die Gesellschaft (soziale Kosten) internalisiert und die Verursachenden steuerlich belastet, um Anreize für die Vermeidung von Emissionen zu setzen. Die Häfen haben in den letzten Jahren erhebliche Initiativen zur nachhaltigen Entwicklung ergriffen, allerdings sind die Herausforderungen zur Erfüllung des Klimaabkommens weiterhin immens.
